# Effective interventions to increase representation of under-served groups in randomised trials in UK and Ireland: a scoping literature review

**DOI:** 10.3310/nihropenres.13524.1

**Published:** 2024-03-25

**Authors:** Katie Biggs, Caroline Dix, Frances Shiely, Shaun Treweek, Victoria Shepherd, Athene Lane, Heidi Green, Talia Isaacs, Andrew Willis, Cindy Cooper

**Affiliations:** 1 Clinical Trials Research Unit, Division of Population Health, The University of Sheffield, Sheffield, England, S1 4DA, UK; 2 HRB Clinical Research Facility and School of Public Health, University College Cork, Cork, County Cork, T12 WE28, Ireland; 3 Health Services Research Unit, University of Aberdeen, Aberdeen, Scotland, AB25 2ZD, UK; 4 Centre for Trials Research, Cardiff University, Cardiff, Wales, CF14 4YS, UK; 5 Bristol Trials Centre, University of Bristol, Bristol, England, BS8 1NU, UK; 6 COUCH Health, Manchester, M1 3HF, UK; 7 Institute of Education (IOE), Faculty of Education and Society, University College London, London, England, WC1H 0NS, UK

**Keywords:** Under-served groups, inclusion, clinical trials, recruitment, retention

## Abstract

**Background:**

Participants in clinical trials often do not reflect the populations that could benefit from the treatments being investigated. There are known barriers to trial participation for under-served groups, but limited evidence on strategies to alleviate these barriers to improve representation. This scoping review aimed to identify effective interventions and design features that improve the representation `of under-served groups in trials, focusing on the UK and Ireland.

**Methods:**

We included methodological research studies that reported interventions to improve representation of ethnic minority groups, socioeconomically disadvantaged groups, older people, or those with impaired capacity to consent to randomised controlled trials, conducted in the UK and Ireland, published between 2000–2021. Systematic searches were conducted in November 2021 and data were independently extracted by two authors and narratively synthesised.

**Results:**

Seven studies were included: one randomised controlled study embedded in five trials, one mixed-methods study, and five studies reporting ‘lessons learnt’ from one trial. We categorised the 47 reported interventions or strategies into nine broad themes: Recruitment sites, recruitment settings, community engagement, and communication with participants, incentives, inclusion criteria, flexibility, patient documentation, and the consent process. Only 28/47 interventions were evaluated, 23 of which were comparison of recruitment pathways.

The randomised study found that a £100 incentive mentioned in the invitation letter increased positive responses overall across drug trials in cardiovascular disease and hypertension, but not for older people or those living in the most deprived areas. Invitation letters via GPs and working with communities were reported as successful recruitment pathways in recruiting different under-served populations.

**Conclusions:**

Interventions aiming to improve the recruitment of under-served groups in the UK and Ireland were reported across seven papers, but their effectiveness was rarely rigorously evaluated. Included studies were context specific. Using a variety of recruitment methods is likely to help achieve a more diverse cohort.

## Plain Language Summary

Not all people are included in medical research, even though they should be. This is especially true for people from certain groups, called ‘under-served groups’, and there has been a recent focus on improving inclusion of these groups in clinical trials. Researchers have developed tools to help plan studies that include under-served groups. These tools help researchers to consider who should be included in a study, identify barriers to including certain groups, and develop strategies to overcome those barriers.

A team of researchers reviewed existing research to see what strategies have been successful in increasing the participation of under-served groups in clinical trials in the UK and Ireland. They looked at ethnic minority groups, people experiencing disadvantages in income, education, housing and/or employment (socioeconomic disadvantage), older people, and people with limited decision-making abilities (impaired capacity to consent). They found seven studies that provided helpful insights. Two of these studies were designed specifically to test different methods for recruiting under-served groups. The other five studies were based on lessons learned from trials that included these groups.

One study found that offering a £100 incentive in recruitment letters led to an increase in responses, but not specifically for older people or people experiencing socioeconomic disadvantage. Another study found that letters from general practitioners were most effective for recruiting older adults. One study found that community engagement was successful for recruiting an ethnic minority group.

Overall, the review concluded that there is little evidence for strategies that will increase the inclusion of under-served groups in clinical trials in the UK and Ireland. However, there are several promising approaches that could overcome barriers and make research more inclusive.

## Introduction

Participants in clinical trials often do not reflect the populations that could benefit from the treatments being investigated. For example, in the UK and Ireland, COVID-19 has been shown to disproportionally affect older people and those from ethnic minority groups in the UK, and despite this knowledge, these groups were underrepresented in COVID-19 medical research ( Borno *et al*., 2020; Murali *et al*., 2023; Treweek *et al*., 2020b; Veronese *et al*., 2021). Trials leading to drug approval have been shown to underrepresent older people ( Ruiter *et al*., 2019) and ethnic minorities ( Loree *et al*., 2019) and there is a body of work focused on people with impaired capacity to consent ( Shepherd *et al*., 2019a; Shepherd *et al*., 2019b; Shepherd *et al*., 2019c) that highlights the methodological, structural and systemic barriers to their inclusion in trials ( Shepherd, 2020).

There are several negative consequences to participant populations that do not look like the patients that could ultimately receive the trial’s intervention. For example, the under-served groups may miss out on the opportunity of participating in trials, and trial conclusions cannot with certainty support treatment decisions for those underrepresented in the trial ( Moloney & Shiely, 2022). Clinicians, and regulators, may be reluctant to generalise trial findings to the target population if they are not relevant for their context.

The UK National Institute for Health and Care Research (NIHR) INCLUDE project was commissioned in 2017 to examine the inclusion of under-served groups in clinical research. It identified a range of under-served groups, shown in Table 1, based on demographic factors, social and economic factors, health status factors and disease specific factors which can vary across the types of studies and disease, or condition being studied. Under-served groups are therefore context-specific, and there is no single definition available, nor a comprehensive list of under-served groups. This notwithstanding, the INCLUDE project identified common characteristics of under-served groups, such as sustained lower participation rates in research compared to the population estimates, groups with a high healthcare burden that is not reflected in inclusion in research, and relatively little group response or engagement to interventions that are not accounted for in the research.

**Table 1.** Examples of under-served groups identified in the NIHR INCLUDE project.

Groups by Demographic Factors (Age, Sex, Ethnicity, Education)	Age extremes (e.g. under 18 and over 75) Women of childbearing age Different ethnic minority groups Male/female sex (depending on trial context) LGBTQI+ / sexual orientation Educational disadvantage
Groups by Social and Economic Factors	People in full time employment Socio-economically disadvantaged/ unemployed/ low income Military veterans People in alternative residential circumstances (e.g. migrants, asylum seekers, care homes, prison populations, traveller communities, the homeless and those of no fixed abode) People living in remote areas Religious minorities Carers Language barriers Digital exclusion/disadvantage People who do not attend regular medical appointments People in multiple excluded categories Socially marginalised people Stigmatised populations Looked after children
Groups by Health Status	Mental health conditions People who lack capacity to consent for themselves Cognitive impairment Learning disability People with addictions Pregnant women People with multiple health conditions Physical disabilities Visually/ hearing impaired Too severely ill Smokers Obesity
Groups by Disease Specific Factors	Rare diseases and genetic disease sub-types People in cancer trials with brain metastases

Table adapted from Witham *et al*. (2020).

In relation to high healthcare burden, it is well known that socioeconomic status (historically referred to as social class in the UK) and healthcare inequalities are linked ( The Black report, 1980). Despite the rise of welfare states in Europe, these inequalities have remained ( Mackenbach, 2019) and, in fact, the association between socioeconomic status, education and health has increased, making people experiencing socioeconomic disadvantage an important under-served group to consider. Socioeconomic disadvantage in cancer research is linked with lower access to trials and worse outcomes when they are included ( Sharrocks *et al*., 2014). Linked to burden and inequalities is intersectionality, a framework that recognises how being a member of more than one marginalised group can intersect and interact, leading to unique experiences of discrimination or privilege, and in inequalities in healthcare ( Kelly-Brown *et al*., 2022; Samra & Hankivsky, 2021).

The INCLUDE project produced a roadmap ( Witham *et al*., 2020) which identifies time points for potential intervention over the lifetime of a trial. This illustrates how researchers, funders, ethics committees, delivery teams, participants, patients, public, and analysts can work together to successfully deliver research that is inclusive and sensitive to the needs of under-served groups.

### Barriers

Several barriers to recruitment of under-served populations to trials were identified in the NIHR INCLUDE project ( NIHR, 2020); barriers relating to physical disability, lack of effective incentives, lack of interest in research, negative financial impact, poor consent procedures, risk perception, burden and support required for participation. Other research has identified barriers specific to certain groups, for example, those lacking capacity to consent have legal barriers surrounding providing consent ( Shepherd, 2020); Black African American communities are found to have less trust in research than white Americans ( Corbie-smith *et al*., 2002); and South Asian communities in the UK experience barriers relating to health care use, language and the importance of family and community ( Brown *et al*., 2014; Hussain-Gambles *et al*., 2004).

### Trial design

The NIHR INCLUDE Frameworks ( Gardner *et al*., 2022; Shepherd *et al*., 2022; Treweek *et al*., 2021) guide researchers through important questions when designing trials to help researchers think about what can be done to reduce barriers for groups that are under-served due to their ethnicity (including culture, faith, and language), experience of socioeconomic disadvantage, or due to their impaired capacity to consent. There is also guidance for including older people in health and social care research on the Trial Forge website. This might involve adjustments to trial design or include specific interventions to improve engagement between the trial team and specific ethnic minority groups. NIHR funding streams now emphasise more the need for consideration of inclusivity, but research teams and Clinical Trials Units (CTUs) may lack experience in this area and not know what interventions to put in place to improve inclusion in trials.

### Strategies for improving inclusion in trials

Methodological interventions have been suggested to improve representation of under-served groups in the literature ( Ismail *et al*., 2014; Liljas *et al*., 2019; Shepherd, 2021; Velzke & Baumann, 2017), and previous reviews on improving recruitment of under-served groups to trials from international studies ( Bodicoat *et al*., 2021; UyBico *et al*., 2007) highlight the need for effective interventions in this area. However, the variable methodological rigor and evidence gaps indicate that further research is necessary to address this issue comprehensively. A recent review of international research ( Bodicoat *et al*., 2021) identified evidence that cultural competency training for recruiting staff and personalising communication improved representation, but no strategy was effective across trials or populations, and they recommended a multi-faceted approach to the recruitment of under-served groups. Masood *et al*. (2019) undertook a review of trials that aimed to recruit South Asian populations in the UK and identified the following strategies: Adaptation of screening and outcome measures, culturally specific recruitment training, working with religious leaders, collaborating with ethnic community organisations, self-referrals and assistance from family and carers, recruitment sites in diverse areas, multilingual written invitations, translation of the participant information sheets, tape recorded participant information, choice of interview location, follow-up arrangements, linguistic matching, ethnic matching, gender matching and awareness of the cultural practices and norms. However, these strategies were not evaluated. Prior research tells us that that recruitment strategies are not recorded or reported in most trials making evaluation of used strategies impossible ( O’Sullivan Greene & Shiely, 2022).

Trials aimed at general populations based on disease are more common than those focussed on a specific under-served group, and due to the number, breadth and intersectionality of under-served groups, trialists need to consider a range of under-served groups to improve inclusion in trials.

A scoping review was chosen to identify existing methodological interventions across a range of under-served groups, trial types and using various methods of evaluation to provide information on their effectiveness.

***Objective.*** The objective of this scoping review is to identify, report, and evaluate the effectiveness of interventions aiming to improve representation of four under-served groups in trials in the UK and Ireland, as described below.

## Methods

### Protocol and registration

A protocol for the ACCESS project was published on the Sheffield CTRU website prior to the start of the final searches: https://www.sheffield.ac.uk/scharr/research/

This scoping review forms work package 1 of the work described in the protocol.

### Patient and Public Involvement

Patients and public were not involved in the scoping review but were involved in the later stages of the ACCESS project where the results of the scoping review were presented and used to stimulate further discussion around inclusive trial methodology. 

### Scoping review strategy

A scoping review was conducted according to Joanna Briggs Institute (JBI) methodology guidance for scoping reviews ( Peters *et al*., 2020) to ensure a rigorous, transparent and trustworthy evidence synthesis to explore and summarise the literature across a range of under-served groups and trial design. A literature review was undertaken on trials that evaluated interventions to improve the representation of under-served groups. An initial scoping exercise using the Online Resource for Research in Clinical triAls database ( ORRCA) was undertaken to explore the relevant literature on improving representation of under-served groups in clinical trials. Following this, the search strategy was developed in consultation with the collaborator group. Based on the scoping exercise and collaborator experience, the search focussed on the following under-served groups: minority ethnic groups, socioeconomically disadvantaged groups, those with impaired capacity to consent and older people. We focussed on four under-served groups, as commonality and intersectionality of under-served groups means focussing on one under-served group is unlikely to be sufficient in making trials more inclusive.

### Eligibility criteria

Inclusion criteria were:

- Types of studies: All types exploring methods of recruitment to randomised controlled trials (RCTs). Not RCT reports unless there was a methodological focus on recruitment and retention of under-served groups in the paper.
- Concept of interest: Participation in RCTs.
- Participants: Those from ethnic minority backgrounds, those experiencing socioeconomic disadvantage, older people and those that lack capacity to consent.
- Type of intervention: Any interventions used to improve the recruitment of under-served groups in RCTs.
- Type of outcome measures: Any measure of effectiveness adopted by the authors, e.g. participant recruitment, participant knowledge.
- Geographical area: United Kingdom and Ireland.
- Years: 2000–2021.
- Language: English.
- Output type: Primary research papers.

Exclusion criteria were:

- RCT reports that did not focus on recruitment or retention of under-served groups in the title or abstract.
- Studies that were about recruitment to qualitative research, quantitative non-RCTs or Patient and Public Involvement & Engagement (PPIE) activities.
- Review articles, reports, commentaries, and studies not focussed on those from ethnic minority backgrounds, those experiencing socioeconomic disadvantage, older people and those lacking the capacity to consent.

### Search strategy

The search strategy (Extended data: Appendix 1 ( Biggs, 2024)) was comprised of search string relating to randomised controlled trials, a search string for papers conducted in the UK and Ireland, and a string related to recruitment, retention and inclusion. Search strategies were developed for each under-served group and combined with these.

### Information sources

CD searched PubMed for published papers on 29 ^th^ November 2021. We also included one paper identified through a preparatory search whilst developing the search string, this was not identified in the final search due to the addition of the RCT and UK and Ireland filters.

### Quality of the included studies

We did not perform a formal assessment of the quality of included studies in line with the recommendations for scoping reviews ( Peters *et al*., 2020).

### Study selection process

The titles and abstracts of the papers were reviewed independently by KB and CD using Mendeley. The full text publications were retrieved and screened by both KB and CD, and they had regular meetings to discuss the interventions being identified and data extraction.

### Data items and charting

The following data were extracted from the included papers: Author, date, background/conclusions, methods, population, researchers’ definition of the population, trial description, disease area, host trial intervention, methodological intervention, rationale for intervention, implementation, recruitment figures, retention figures, qualitative findings, costs of the intervention, author discussion around effectiveness of interventions, and author recommendations. Data were extracted into a spreadsheet for all included papers by KB and CD independently and discussed.

### Synthesis of results

The methodological interventions identified during data extraction were explored by KB and organised into themes based on categories in a previous UK review ( Masood *et al*., 2019). The interventions and themes identified in the included papers were discussed at a collaborator meeting. The previous categories were related to interventions to recruit an ethnic minority group, therefore some needed to be amended or widened to cover the additional under-served groups included in this review, some of the categories were split to provide additional detail and additional categories were added. 

## Results

**Figure 1.**
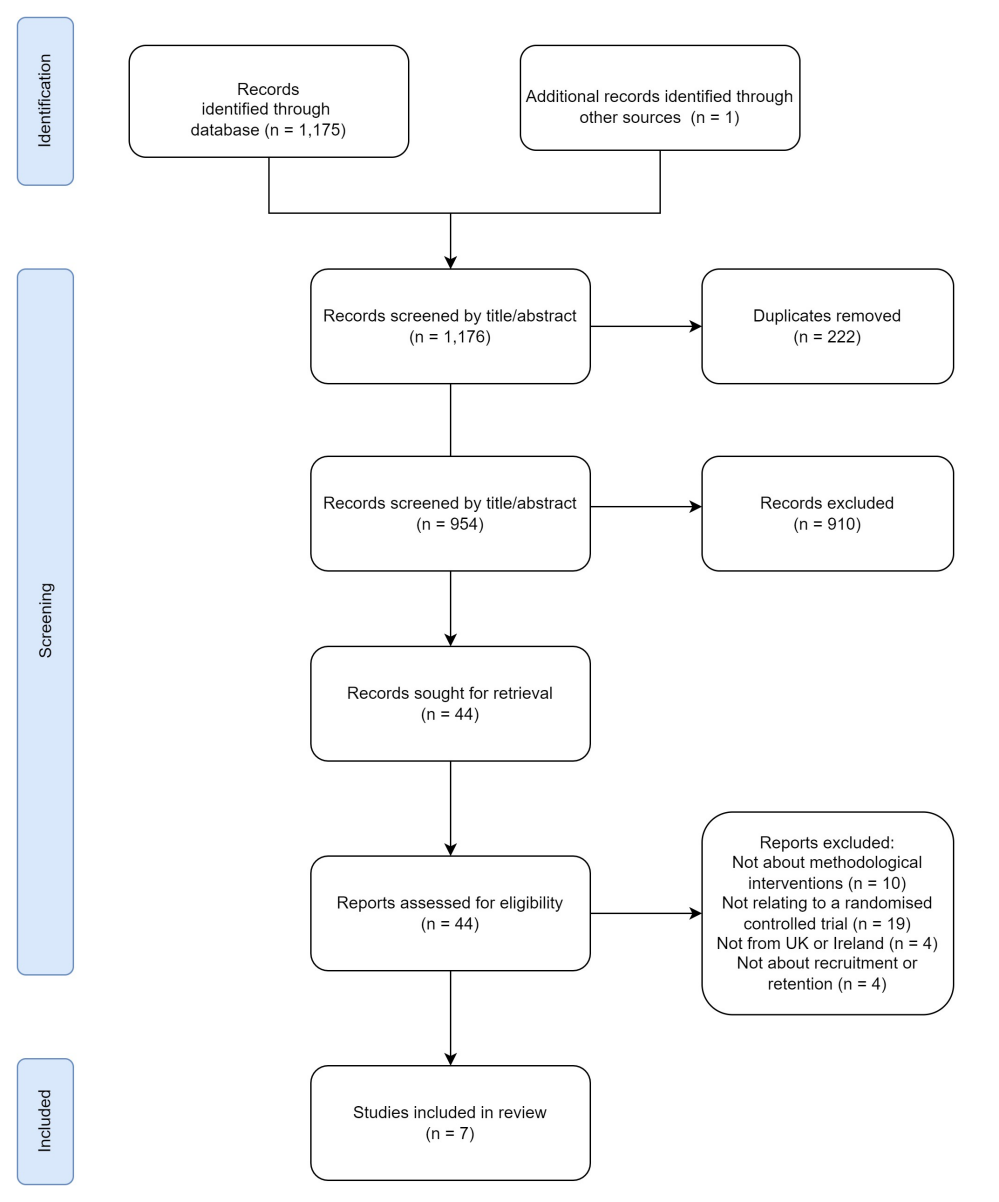
PRISMA diagram.

A summary of the literature search is presented in the PRISMA diagram ( Figure 1). There were 1,176 papers initially identified from the search and other sources (954 papers after removal of duplicates). Following application of the inclusion and exclusion criteria, 44 papers underwent full-text screening. The full papers were then screened and 37 were excluded. Seven papers were ultimately included in the full review.

### Included paper characteristics

Characteristics of the included studies are presented in Table 2, this includes details of the trial(s) the methodological interventions were evaluated in or for.

**Table 2.** Included studies’ characteristics and trial characteristics.

Lead author, date	Author’s definition of population (under-served category)	Disease area	Aims/purpose	Methods	Trial description / design	Trial intervention(s)	Number of participants	Interventions discussed
Agnew *et al*., 2013	Community-dwelling older women 60 and over (Older people)	Urinary incontinence	To evaluate engaging community organisations for the recruitment of community-dwelling older women with incontinence to a randomised controlled trial.	Lessons learnt from one trial (retrospective)	Four-arm cluster randomised controlled trial (RCT)	Three continence promotion workshops	667 women attended community workshops; 192 randomised	Workshops prior to screening and enrolment
Douglas *et al*., 2011	South Asians of Indian- or Pakistani-origin, aged 35 years or over, without diabetes (Ethnic minority group)	(Pre) Type 2 Diabetes	To share their experience of recruiting South Asian participants to the PODOSA (Prevention of Diabetes and Obesity in South Asians) trial.	Lessons learnt from one trial (retrospective)	Two-arm cluster RCT	A family focussed, home-based, lifestyle-intervention, working with a trained dietitian over three years	2,089 participants referred through all routes; 171 randomised	Compared different recruitment settings.
Forster *et al*., 2010	65–85 years (Older people)	Nutrition - no specific disease or condition	To describe the recruitment strategies used to identify older adults for recruitment to a 6-month randomised controlled dietary intervention trial (The FIT study).	Lessons learnt from one trial (retrospective)	Three-arm RCT	Micronutrient tablet, or dietary intervention for 3 months, where participants were given approximately £15 worth of food each week	7,482 invitation letters sent + other methods of recruitment; 217 randomised	Compared different recruitment settings.
Jayes & Palmer, 2014	Participants with a mild, moderate, or severe comprehension impairment as determined by the Comprehensive Aphasia Test (People with impaired capacity to consent)	Aphasia	To evaluate the Consent Support Tool (CST), which aims “to identify the optimum format in which to present research information to people with different severities of aphasia, in order to support the informed consent process.”	Mixed methods study on trial recruitment tool	Not related to a specific trial but about informed consent to trials. Developed for Little CACTUS trial – two arm-pilot RCT	Computer speech and language therapy	14 participants (recruited from the Little CACTUS trial participants, N=34)	Evaluated the Consent Support Tool (CST)
Jennings *et al*., 2015	Socially deprived (based on address), age (Older people/ socioeconomically disadvantaged)	Cardiovascular (two trials), hypertension (three trials)	To assess the impact of a £100 incentive payment mentioned in the invitation letter, and whether it attracted older and more socially deprived patients.	2-arm SWAT RCT across 5 trials	SWAT across 5 trials: Two-arm CTIMPS	Drug	1,015 invitation letters sent; 202 randomised	£100 incentive mentioned in the invitation letter
Kolovou *et al.*, 2020	Sites in areas of high deprivation (Socioeconomically disadvantaged)	Cancer awareness for general public	To report an example of successful recruitment and retention to the ABACus3 trial in a trial targeting a socioeconomically disadvantaged population.	Lessons learnt from one trial (retrospective)	Two-arm, RCT, with process evaluation and interviews	‘The Health Check’ - Facilitated cancer awareness intervention (delivered in community venues)	448 individuals were assessed for eligibility in community settings, 234 randomised	Discussion of methods used in the successful recruitment of an under-served population.
Withall *et al*., 2020	Community-dwelling adults, aged 65 years and older (Older people)	Ambulatory patients at risk of mobility disability	To report on the cost, strategies, feasibility and ‘lessons learned’ from recruiting at-risk community-dwelling older adults to the REACT trial.	Lessons learnt from one trial (retrospective)	Two-arm, parallel-group RCT. including an internal pilot, process and economic evaluations.	A group-based exercise and socio-educational intervention	25,559 invitation letters sent; 777 randomised	Compared different recruitment settings.

***Under-served population.*** All seven included papers described interventions to improve recruitment of an under-served group to clinical trials in the UK or Ireland and evaluated them. One trial concerned the recruitment of South Asian participants, two trials concerned the recruitment of socioeconomically disadvantaged participants, with one of these also focused on older people. Three trials were solely focussed on older people and one trial was concerned with recruiting people with aphasia (who may have impaired capacity to consent).

***Study methods.*** The included papers include one randomised evaluation, a randomised evaluation across five UK trials looking at the £100 incentive and non-randomised evaluation of the Consent Support Tool, which was a well-designed mixed methods study of a tool to support recruitment. The remaining papers retrospectively report recruitment or retention strategies with no comparative evaluation. These five papers were ‘lesson learnt’ papers reporting on one trial. They either compared recruitment settings or discussed a range of methods used in their trial and apart from where recruitment settings were compared, the interventions discussed did not have a comparator.

### Trial characteristics

***Trial design.*** One paper evaluated a methodological intervention across five trials, all two-arm RCTs, and one paper evaluated a tool to be used in a two-arm pilot trial. The remaining papers all reported on methodological interventions from an RCT, one four-arm cluster RCT, one three-arm RCT and three two-arm RCTs.

***Trial population.*** The trials’ populations included participants with cardiovascular conditions, hypertension, and Aphasia, participants with untreated urinary incontinence, pre-diabetes, and populations without an existing diagnosis, such as elderly participants at risk of mobility disability, improving nutrition in older people, and cancer awareness. Number of participants recruited ranged from 34–777.

***Trial interventions.*** Interventions included public health strategies, group-based interventions, speech and language therapy, dietary consultation, nutrition supplements and drugs. 

### Interventions to improve recruitment of under-served groups

The papers reporting on ‘lessons learnt’ from a trial ( Agnew *et al*., 2013; Douglas *et al*., 2011; Forster *et al*., 2010; Kolovou *et al*., 2020; Withall *et al*., 2020) discussed more than one methodological intervention, with 48 discussed in total. Each intervention is listed in Table 3 and categorised into nine main themes.

**Table 3.** Interventions discussed in the papers, and the author’s interpretation.

**Main theme**	**Author, date**	**Intervention to improve recruitment of under-served groups**	**Author’s findings**
Recruitment sites	Agnew *et al*., 2013	Authors made a conscious effort to approach culturally and socio-economically diverse groups of women throughout the United Kingdom.	Reported that included organisations represented a wide socioeconomic and educational base. No socioeconomic or ethnicity data reported.
	Kolovou *et al*., 2020	Recruitment took place in two geographical areas: South and West Yorkshire and Southeast Wales. Within these areas neighbourhoods of high socioeconomic deprivation (10% most deprived or 10–20% most deprived) were identified using national deprivation indices.	More than half of the participants lived in the 10- 20% most deprived areas-. Authors reported that their sample may not be representative ethnic minority communities.
	Withall *et al*., 2020	Three trial sites were chosen that represented urban, suburban, and semirural locations with diverse socioeconomic and ethnic characteristics. Authors over-recruited General Practices (GPs) in diverse areas to allow for an anticipated lower response rate from ethnic groups and the most deprived.	Quintile 1 (most deprived) of the Index of Multiple Deprivation (IMD) = 11.1% compared to 14.3% of the general UK population of over 65-year olds; Quintile 2 = 20.2% recruited, 17.6% in general population. Asian participants = 1.2% recruited, 2.6% in general population. African/Caribbean participants = 3.0% recruited, 1.3% in general population. Caucasian/white participants = 95.1% recruited, 95.5% in general population Other/mixed ethnicities = 0.8% recruited, 0.7% in general population. Male = 33.85% recruited, 45.6% in general population. They reported that targeted efforts could help to recruit more ethnically diverse cohorts.
	Forster *et al*., 2010	Researchers approached GPs in areas of lower socio-economic status first...	No socioeconomic data reported. No further comments in the discussion.
Recruitment approach	Agnew *et al*., 2013	Recruitment of community organisations to hold a workshop to recruit participants. Compared four types of workshops (interactive; self-management; interactive & self-management; control).	No differences in recruitment rate between groups. Authors reported that using community organisations for the recruitment of community-dwelling older women their trial was successful. Authors reported difficulties recruiting community organisations, and work is needed in this area to build relationships.
	Douglas *et al*., 2011	Direct referrals from health care professionals (NHS), primary and secondary care.	Largely unsuccessful, recruited 3% of total referrals (target was 25%).
		Written invitations via GPs to potential participants.	Low success (5.2% of total referrals, target 25%) response rate to letters was resource intensive.
		Written invitation via diabetes register to diabetes patients (to target their relatives).	Unsuccessful, 0 people screed via this method.
		Search of practice lists for patients meeting specific inclusion criteria.	Unsuccessful, 0 people screed via this method.
		Recruitment via research team contacts, self-referrals, and use of the ‘snowball’ effect.	One of three methods, totalling 50% of the recruitment. Author’s reported that word of mouth was particularly successful in Glasgow, and that costs per participant were minimal. The partnerships with the local South Asian organisations and individuals, and referrals by word of mouth from existing participants were the most successful strategy. Snowballing was successful – three recruited participants led to the screening of 140 others.
		Research team recruitment via visits/talks.	One of three methods, totalling 50% of the recruitment. Moderately successful but labour intensive.
		Advertising: Written articles in the press, radio interviews, leaflet and poster distribution, website and e-mail distribution lists.	Not successful in directly enrolling participants.
		Advertising: Ethnic marketing and consultancy company.	Limited success achieved by fieldwork, not mass marketing (1 screened).
		Community organisations and recruiters, assisting with recruitment for small payment.	This is one of 3 settings totalling a target of 50% recruitment. Initially unsuccessful when relying on goodwill, moderately successful when payment offered.
	Kolovou *et al*., 2020	The researchers recruited from a range of healthcare settings in all identified neighbourhoods. Healthcare venues: GP surgeries, community pharmacies.	Author’s reported that the healthcare settings were challenging and time-consuming to approach and set-up They suggested this was due to the hierarchy in communication, and the complex delegation of responsibilities amongst staff in healthcare settings.
		Community venues: libraries, social clubs, sheltered housing, homeless service centre, community centres and churches.	Community settings had higher percentages of unemployed and self-employed participants, education, employment, ethnicity and deprivation did not differ between settings. Using community settings for recruitment (in addition to healthcare venues) allowed for the recruitment of participants who are not regular visitors to healthcare settings.
	Forster *et al*., 2010	Recruitment through GPs. GPs in areas of lower socio-economic status were approached first.	Writing directly to potential participants via GPs was the most successful recruitment strategy (195 participants recruited, 90% of total recruitment).
		Recruitment through Barnsley Metropolitan Borough Council.	3 (1.4%) participants recruited. Authors did not request ethical approval to send reminder letters, but suggested that they may have helped the recruitment rate after the initial contact letter.
		Advertising: Recruitment through posters in community groups and 2 advertisements were placed in the local newspaper.	7 (3.2%) participants recruited. No further comments in discussion.
		Recruitment through interviews about the trial by two local radio stations.	0 participants recruited. No further comments in discussion.
		Recruitment through a stand in a local supermarket ASDA and market.	4 (1.8%) participants recruited. No further comments in discussion.
		Recruitment through presentations to a range of groups including the Women’s Institute and Friendship groups.	1 (0.5%) participant recruited. No further comments in discussion.
		Snowballing via participants.	7 (3.2%) participants recruited. No further comments in discussion.
	Withall *et al*., 2020	Primary care (letters from GPs). GPs were recruited via the UK Clinical Research Network (CRN).	GP practices were the most productive recruitment route (Recruited 682 participants (87.8% of total recruitment)). Some GP practices in diverse areas were already involved in other research that was aiming for a diverse sample and were unable to participate.
		Third-sector organisations: community groups, social enterprises and sheltered housing facilities.	Sheltered housing, recruited = 8, (1.02%) Community partners, recruited =12, (1.5%) Found presentations, relationship building, and meetings with community groups and established partners added only small numbers of participants, while requiring considerable staff resources. But they did find these relationship-based approaches supported recruitment within diverse communities.
		Word-of-mouth, and snowball techniques (friends, relations, or spouses of invitees).	Recruited = 23 (3%). No further comments in discussion.
		Advertising: A supplementary low cost (£726) public relations (PR) campaign.	5.4% of total recruitment figures. £17.29 cost per recruit. No further comments in discussion.
Community engagement	Kolovou *et al*., 2020	Lay advisors were trained to deliver the intervention and helped with recruitment. To support recruitment they communicated with key stakeholders, identified eligible venues, liaised with local gatekeepers, organised recruitment days, and recruited participants.	The lay advisors thought community recruitment was successful because there was a lot of people visiting the community venues, the visitors had free time on site and were more willing to hear about the trial, and “older visitors” enjoyed talking to the lay advisors. There was no discussion around the impact of the lay advisors delivering the intervention.
	Withall *et al*., 2020	Local community groups, charities, and the public sector facilitated events to explain and discuss the study with their service users and issued written invitations. A close working relationship was established to achieve this.	Not discussed in relation to raising awareness, used also for recruitment (see above).
Communication between study team and participants	Douglas *et al*., 2011	The study employed three South Asian bilingual dietitians, two had extensive work experience in the recruitment area	No discussion around the impact of employing bilingual staff.
	Kolovou *et al*., 2020	Participants were told in advance that the researcher would call from a number from a specific area code.	Not specifically mentioned in discussion. Commented on high retention rates at 2 weeks (90.5%) and 6 months (85.0%).
		Participants were given a general timeframe for their follow-ups.	
		Emphasis was placed on the lay advisors’ affiliation with the University (to increase trust).	
	Forster *et al*., 2010	Strategies were put in place to help participants with reading and writing difficulties, such as getting help from partners and relatives and researchers.	This support required extra time which had to be planned for. The authors found that encouragement and reassurance were especially important in help in participants complete the task.
	Withall *et al*., 2020	Provided funds for translators.	Figures around translation not reported.
		Researchers aimed to build rapport and trust during telephone and face-to-face contacts. Telephone contact was prompt (within 3–4 days) and friendly.	Research staff thought this was one of the critical success factors.
Incentives	Jennings *et al*., 2015	£100 incentive mentioned in invitation letter (not mentioned in letter for control group, but still given to participants).	Mentioning the £100 incentive did lead to more people to respond positively to an invitation letter (6.9% increase) and resulted in slightly more randomised patients, however, this effect was marginal. The incentive payment did not attract older or participants living in the most deprived areas. Even where a significant improvement was observed, it was not a cost-effective recruitment method.
	Kolovou *et al*., 2020	Participants were offered a £10 High Street shopping voucher after completing their baseline questionnaires and a £5 voucher for completing the 6-month follow-up.	Authors reported high retention rates of recruited participants at the 2-weeks (90.5%) and 6-months (85.0%) follow-up points. Participants were offered a financial incentive at recruitment that may have impacted on their willingness to take part. The lay advisors highlighted the value of the participant’s contribution to research by participating, which authors thought may have improved trust and reciprocity.
	Forster *et al*., 2010	Participants were notified about a £100 completion bonus after displaying initial interest in the study..	8/217 people dropped out overall. Authors thought the incentive may have helped with retention.
	Withall *et al*., 2020	Participants were informed at the time of recruitment that they would receive shopping vouchers - £15 for each of the 6-, 12-, and 24-month follow-up visits.	Retention not reported. Not discussed further.
Flexibility	Withall *et al*., 2020	Delivery of multiple face-to-face screening sessions, the research team gave participants date/time choices and reimbursed travel expenses for assessments.	Retention figures not reported but authors reported that trial staff thought this was one of the critical success factors.
	Kolovou *et al*., 2020	Flexibility in recruitment methods: pre-booked appointments or opportunistic recruitment. Lay advisors adopted opportunistic recruitment, approaching individuals in a community or healthcare setting.	Recruitment was restricted to weekdays and working hours which may have impacted on the recruitment of working adults. Authors noted that community venue staff may have inadvertently biased recruitment.
		Participants were asked their preferred time (weekend/weekday,morning/afternoon/evening) and method of contact (phone call, text message, e-mail, post) for their follow-ups.	Authors reported high retention rates at the 2-weeks (90.5%) and 6-months (85.0%) follow-up. A personalised flexible follow-up approach may have allowed for added trust and reciprocity between the participants and the researcher lay advisors.
Participant-facing written materials	Kolovou *et al*., 2020	All patient-facing study materials were written in line with national average literacy levels and were reviewed by the trial’s Patient and Public Involvement group prior to recruitment.	Retention rates were high, indicating people were fully informed of what the trial involved. Authors reported that a study limitation was the lack of translated materials and language support, as this contributed to limited recruitment of people with limited English language.
	Withall *et al*., 2020	Patient documentation: In the pilot, the Participant Information Sheet (PIS) was sent out on receipt of a response/enquiry form but this was changed in the main trial to save time and effort. The invitation letter was changed to provide a much more noticeable required participant profile in a large, prominent text box, and the PIS was sent with the invitation letter.	The response rate to the initial invitation letter was 8% lower in the main trial than the pilot study but a much higher proportion of responders progressed through telephone screening to face-to-face assessments compared to the pilot study (43.3% vs 27.2%), indicating that more eligible candidates responded. Authors reported that making eligibility clear, prominent and in plain language helped with this.
Inclusion criteria	Withall *et al*., 2020	The change of trial’s inclusion criteria to SPPB 4–9 from 4–8 widened the participant pool to include frail and prefrail populations were eligible for the trial.	Figures before and after this change were not provided. Authors reported that this positively impacted inclusion rates at the face-to-face screenings.
Consent process	Jayes & Palmer, 2014	Consent Support Tool (CST). A tool to facilitate the involvement of people with communication disorders.	The results show that the CST can be used to accurately identify the best information style for participants.

The interventions identified in the included trials are listed below, grouped into themes guided by the type of intervention or strategy and by previous reviews. There was considerable overlap across the papers, particularly in relation to recruitment settings.

Two studies discussed recruitment sites, which refers to where the sites are geographically, and how diverse the areas are, choosing sites with the targeted population demographics ( Kolovou *et al*., 2020; Withall *et al*., 2020) and one study targeted culturally and socio-economically diverse groups of women ( Agnew *et al*., 2013).

Five studies reported on one or more recruitment settings, the recruitment pathway and where participants were recruited from. Settings included:

- NHS referrals, lists and venues, including GPs ( Douglas *et al*., 2011; Forster *et al*., 2010; Kolovou *et al*., 2020; Withall *et al*., 2020),
- community venues ( Agnew *et al*., 2013; Douglas *et al*., 2011; Forster *et al*., 2010; Kolovou *et al*., 2020; Withall *et al*., 2020),
- housing and homeless services ( Kolovou *et al*., 2020; Withall *et al*., 2020),
- researcher talks ( Douglas *et al*., 2011; Forster *et al*., 2010),
- advertising (newspapers, websites, social media, radio, posters) ( Douglas *et al*., 2011; Forster *et al*., 2010; Withall *et al*., 2020) and,
- self-referral, ‘snowballing’/ word-of-mouth ( Douglas *et al*., 2011; Forster *et al*., 2010; Withall *et al*., 2020).

One paper used community engagement and lay advisors for delivery of the intervention ( Kolovou *et al*., 2020) and another used community engagement to raise awareness of the health condition and research ( Withall *et al*., 2020)

Communication between researchers and participants was mentioned in four papers: one employed bilingual staff to deliver the intervention and recruit ( Douglas *et al*., 2011), one highlighted affiliation with the university and provided further information to improve trust ( Kolovou *et al*., 2020), one allowed/provided support to complete questionnaires ( Forster *et al*., 2010) and one provided funds for translators and built rapport with participants through friendly telephone and face-to-face contacts ( Withall *et al*., 2020).

Financial incentives were discussed in four papers ( Forster *et al*., 2010; Jennings *et al*., 2015; Kolovou *et al*., 2020; Withall *et al*., 2020). Flexibility in appointments for recruitment ( Kolovou *et al*., 2020) and follow-up ( Kolovou *et al*., 2020; Withall *et al*., 2020) was discussed in two papers. Two papers mentioned the design of their invitation letter ( Withall *et al*., 2020), or patient information sheet, where they had patient and public input and adhered to national average literacy levels ( Kolovou *et al*., 2020). Widening inclusion to more frail patients to increase the recruitment of older people was reported in one paper ( Withall *et al*., 2020) and the Consent Support Tool was evaluated to see whether the tool could determine the level of information needed for the participant to consent ( Jayes & Palmer, 2014).

These interventions were grouped into the following categories, adapted from categories in two previous reviews ( Bodicoat *et al*., 2021; Masood *et al*., 2019): Recruitment sites and settings, Community engagement, Communication between study team and participants, Incentives, Widening the inclusion criteria, Flexibility, Patient documentation, and Consent process. Categories that were not in the previous reviews were: incentives, widening the inclusion criteria, advertising as a recruitment method, and using the Consent Support Tool to facilitate the consent process.

Table 3 provides further details of the interventions described in each paper, and the authors’ evaluation of the intervention. A table of the main and sub-themes identified is in Extended data: Appendix 2 ( Biggs, 2024).

### Evaluation of methodological interventions to improve recruitment of under-served groups

Not all interventions were evaluated in the included papers; six papers reported recruitment data, with five of these reporting the impact of different recruitment settings on recruitment. One paper found no difference in recruitment between four types of workshops (varying in content and participant interaction) ( Agnew *et al*., 2013), and one showed few differences in ethnicity, education or employment between participants recruited from a healthcare or a community setting ( Kolovou *et al*., 2020). Three other papers compared a number of settings, with one reporting 73% of their screened participants being recruited via the research team contacts, snowball sampling, talks and through community organisations, and these were the most successful methods for recruiting South Asian participants ( Douglas *et al*., 2011). Two studies reported that writing to participants via their GPs was the most successful in recruiting older participants ( Forster *et al*., 2010; Withall *et al*., 2020), with one of these pointing out that more targeted efforts might increase ethnic and SE diversity ( Withall *et al*., 2020).

Jennings *et al.* (2015) used an RCT to evaluate their methodological intervention across five trials. The authors found a 6.9% increase in positive responses to the invitation letter when the £100 incentive was mentioned, but this did not affect the age of those responding or increase the number of socioeconomically disadvantaged participants. Other studies that mentioned an incentive did not assess them, though one mentioned that it may have contributed to recruitment ( Kolovou *et al*., 2020) and the other that it may have contributed to high retention rates ( Forster *et al*., 2010). These two trials were the only ones that reported retention rates ( Forster *et al*., 2010; Kolovou *et al*., 2020), which were high in both.

Jayes & Palmer (2014) did not evaluate their intervention within an existing trial but used a mixed methods approach to evaluate the Consent Support Tool (CST) that would be used in trial recruitment. They found that the tool successfully identifies the appropriate information to give the participant based on their aphasia and can be used in the trial consent process.

One lessons learnt paper ( Kolovou *et al*., 2020) asked lay advisors about their experience of recruitment. They reported that recruitment was successful due to higher footfall in the community venues, and potential participants having time to ask questions and enjoying discussing the research. Lay advisors reported that minimising burden for recruiting centres was helpful in recruiting venues, but not being able to pay for venue hire was a barrier. Another study ( Withall *et al*., 2020) eliciting research staff’s views about recruitment methods reported that friendly contact, rapport building, flexible screening, follow-up appointments and reimbursement for travel were key to the success of the study. 

The other interventions were not formally evaluated across the studies: recruitment sites, design of patient materials, patient and public involvement (PPI), employing bilingual staff, flexibility in recruitment appointments, flexible follow-up, and communication between study team and participants. However, Withall *et al*. (2020) reported that the following elements were key to successful recruitment of their elderly population: invitations and advertising using lay language and providing a good definition of the study, amending inclusion criteria to more frail participants, and prompt and friendly contact to build rapport between researchers and participants. They also commented that although talks at community venues were resource intensive and only recruited a few participants, they supported recruitment of diverse participants.

Although not formally evaluated, three studies reported that involvement with community groups helped with recruitment ( Douglas *et al*., 2011) and there were recommendations to start this work as early as possible, developing a relationship with community leaders who can access, via networks, newsletters or venues, the underserved people you need.

In addition to the findings above, three papers provided further recommendations for recruiting and retaining their included populations. In relation to recruiting elderly populations, Withall *et al*. (2020) recommends more accurate targeting to improve response rates and reduce costs, but that in large-scale RCTs, these should be in addition to large-scale approaches, such as mailouts. They found their internal pilot useful in fine-tuning recruitment methods and that building rapport and trust was important as potential participants passed through the screening process. Forster *et al*. (2010) recommends minimising respondent burden to maximise response rates at the recruitment stage of a trial and they felt support for completion of participant documentation, either from friends and family, or researchers, and reassurance were important in helping participants complete tasks. In relation to recruiting adults experiencing socioeconomic disadvantage, Kolovou *et al*. (2020) states that future studies might benefit from community engagement and recruitment through communities and local gatekeepers. They suggested that more work is needed on how to include groups who were underrepresented in their trial: men, ethnic minority communities and adults from socioeconomically disadvantaged areas in part- or full-time employment.

## Discussion

### What did we find?

Seven papers were identified for inclusion in this scoping review, demonstrating that published empirical evidence exists to support trialists in the UK and Ireland to improve representation of four key under-served groups: people from minoritised ethnic communities, people experiencing socioeconomic disadvantage, older people, and people with impaired capacity to consent.

The seven included papers reported various interventions that we categorised into nine broad themes, six of which had been reported in previous UK reviews: Recruitment sites, recruitment settings, community engagement, communication between study team and participants, flexibility, and patient documentation. Interventions specific to the papers included in this scoping review were: incentives, consent processes, widening inclusion criteria, and advertising campaigns. Only the financial incentive, consent support tool and recruitment settings were evaluated.

The only randomised evaluation was of a £100 incentive mentioned in the in the invitation letter, which improved positive response rates to the invitation letter, although there were no differences in the age or number of people from the most deprived areas between those who were offered the incentive, and those not ( Jennings *et al*., 2015). There was only one other pre-planned evaluation ( Jayes & Palmer, 2014) which showed the Consent support Tool could be used in the consent process for people with communication issues.

### What does this mean for trialists in the UK and Ireland?

The lack of evaluation identified in the review means we cannot draw firm conclusions about successful interventions for improving inclusion in trials for these under-served groups. The papers suggest that having different recruitment pathways can be helpful in recruiting diverse under-served groups. Community recruitment can be beneficial for some under-served groups, but is resource intensive, and consent support processes can be used to aid consent. 

The studies are context-specific, and interventions shown to be effective for one under-served group may not be effective for others. Several settings and under-served groups were included in this review, and most trials adopted more than one recruitment pathway. As a minimum, it would be helpful for trialists to report the recruitment rates by under-served group if using more than one recruitment pathway or method in reports. Pre-planned assessments of recruitment and retention methods are encouraged so that good (and bad) practice can be shared and learnt from. 

### How does this compare to previous reviews?

This scoping review shows that limited evidence is available when assessing what interventions can be done to improve the recruitment of four under-served groups in the UK and Ireland but that letters via the GP seem effective for recruitment of older people, and community engagement and lay advisors can aid recruitment of South Asian populations. A previous review reported strategies in the US for improving inclusion but stated methodological rigor was variable and there were significant evidence gaps ( UyBico *et al*., 2007). A more recent review ( Bodicoat *et al*., 2021), including papers from the US, which reported that no strategy for recruitment was successful across populations and that several methods should be used when recruiting under-served groups. This is also evident in this review where comparisons of recruitment methods were made, with each method recruiting some people and one author highlighting that targeted efforts in recruiting older participants might also improve the recruitment of ethnic minorities.

A previous UK-based review examined the recruitment of South Asians to trials and reported several strategies for recruitment, though did not evaluate them ( Masood *et al*., 2019). One strategy reported was to use lay advisors from the community to help with recruitment, which Douglas ( Douglas *et al*., 2011) found that community engagement and lay advisors were the best method of recruitment of the South Asian community in their trial.

### Strengths and limitations

The main strength of our review is its focus. A number of previous reviews have focussed on ethnic minority groups, and on more general ‘under-served’ ( Bodicoat *et al*., 2021), ‘vulnerable’ ( UyBico *et al*., 2007) or ‘socially, culturally, or financially disadvantaged’ groups ( Bonevski *et al*., 2014), whereas we chose to focus this review on four specific under-served groups. We identified papers specifically relating to ethnic minority groups, socioeconomic disadvantage, older people and people with impaired capacity to consent, which allowed us to explore interventions used in different populations. This is important when considering more than one under-served group, which trialists should be doing. There are some common features to the barriers for under-served groups, and further barriers due to intersectionality of under-served groups, that trialists should work to overcome. We also focussed searches to the UK and Ireland to make the findings relevant to the healthcare system, and population in these countries. We used methods to support the systematic approach ( Peters *et al*., 2020) and have reported in line with the PRISMA-SCR reporting guidance for scoping reviews ( Tricco *et al*., 2018).

The review only identified seven papers, which could be due using only one database, and one paper was identified from other sources. Limiting the scoping review to the UK and Ireland limited the number of relevant papers, as there is a work relating to improving inclusivity in trials outside the UK and Ireland ( Bonevski *et al*., 2014; UyBico *et al*., 2007) which could provide important lessons even if effectiveness could not be translated to a UK and Ireland setting.

We did not include patient and public involvement in the review, so we are interpreting these papers as health researchers, and although some of the researchers may also be members of under-served groups, we did not focus on including lived experience in developing the question for review or interpreting the findings. We are involving PPI in the rest of the ACCESS project.

The interventions identified in this review were included and discussed by the paper authors as they were considered effective in recruiting or retaining their target populations, but there was often no assessment of effectiveness undertaken.

The papers were included due to their focus on a particular under-served group, but the older populations were not necessarily considered as an under-served group in the paper. One of the trials recruiting older people had an age limit of 85, and so excluded part of the older population that is often under-served.

### Recommendations and future research

There is a clear need for the interventions undertaken by trial teams with the aim of improving the recruitment of under-served groups to trials to be evaluated. Without rigorous evaluation, trialists are undoubtedly investing time and money into methods that either 1) do not have an effect, 2) have a harmful effect(s) that remains unreported and/or 3) have a beneficial effect(s) that again, remains unreported. Lack of evaluation and reporting means that others cannot build on potential successes to both replicate evaluations in other trial contexts and fine-tune interventions to optimise their effects. Ultimately, this contributes to research waste.

In line with Brown’s 2014 review, we recommend undertaking nested methodological studies within randomised controlled trials to provide this evidence. There are a number of Studies Within A Trial (SWAT ( Treweek *et al*., 2018; Treweek *et al*., 2020a)) listed on the SWAT repository ( https://www.qub.ac.uk/sites/), that could take account of the recruitment of under-served groups by collecting and reporting the relevant demographics. The evaluation of interventions focused on improving recruitment of under-served groups is one of the top priorities for recruitment methodology research ( Healy *et al*., 2018).

As mentioned above, there is also a need for trialists to report on interventions that are currently being adopted with the aim of improving the diversity of participant populations. Retrospective ‘lessons learnt’ papers, although considered lower evidence than pre-planned evaluations, would be a good start to improving the evidence base for potential interventions and could lead to further effectiveness research in relation to inclusion to trials.

## Conclusions

The review highlights the need for more rigorous evaluation of interventions aimed at improving the recruitment of under-served groups to trials. This includes the need for nested methodological studies within RCTs, and for better reporting of interventions currently being used. While the evidence on interventions for improving recruitment of under-served groups in this review is limited and requires further evaluation, the findings suggest that having multiple recruitment pathways, using community engagement and lay advisors, and employing consent support processes can be beneficial in recruiting under-served groups.

## Data Availability

All underlying data are available as part of the article and no additional source data are required. Open Science Framework: Extended data for ‘Effective interventions to increase representation of under-served groups in randomised trials in UK and Ireland: a scoping literature review’, https://doi.org/10.17605/OSF.IO/9HZNJ ( Biggs, 2024) This project contains the following extended data: Appendix 1: Search strategy.docx Appendix 2: Themes identified from interventions in the included papers.docx Appendix 3: Interventions and evaluation described in the included papers.docx Open Science Framework: PRISMA-ScR checklist for ‘Effective interventions to increase representation of under-served groups in randomised trials in UK and Ireland: a scoping literature review’, https://doi.org/10.17605/OSF.IO/9HZNJ ( Biggs, 2024) Data are available under the terms of the Creative Commons Attribution 4.0 International license (CC-BY 4.0).
