# Supplementary material for: Effective interventions to increase representation of under-served groups in randomised trials in UK and Ireland: a scoping literature review
Source: NIHR Open Res. 2024 Mar 25;4:12. [Version 1] doi: 10.3310/nihropenres.13524.1 (PMC11408910; doi:10.3310/nihropenres.13524.1)
Supplement: Peer review history

# Reviewer report: Reviewer response for version 1

**DOI:** 10.3310/nihropenres.14679.r32719

**Published:** 2024-09-27

**Authors:** Daphne Babalis

**Affiliations:** 1 Imperial College London, London, UK

Thank you for inviting me to review this paper. This is an important topic, the work reported is of a high standard and highlights a crucial area for trialists in the UK and Ireland. The authors should be commended for their work, especially as it is rare to read reviews that focus on more than one under-served group and that acknowledge the significance of intersectionality. A thorough review of 20 years of evidence has identified only 7 papers, emphasizing the ongoing need for robust evidence. 

 As research inclusion is now a condition of funding for many NIHR schemes (announcement may date after drafting of paper), the authors could consider noting this new requirement in their paper and commenting there is an even greater need for evidence-based interventions. If specific funding is going to be ear-marked for research inclusion, we need evidence to underpin this.

 I note a few minor points for consideration below;

 - Introduction/trial design section: mentions “NIHR funding streams now emphasize more the need for consideration of inclusivity,..” As above, it may be useful to update this to say this will soon be a condition of funding for many NIHR schemes: Inclusive research design to become an NIHR condition of funding | NIHR

 - Methods / eligibility criteria: There are several mentions of 'retention' (also elsewhere in the manuscript), however the title and findings of the paper focus on recruitment strategies. Were any strategies for retention considered? If not, I would suggest taking out any references to retention to keep this more focused

 - Discussion / 3rd paragraph / first line: check typo "in the in the invitation letter"

 - A range of terms are used throughout for describing people with lived experience i.e. 'lived experience, PPI, PPIE, patients/public, lay advisors - suggest choosing one term throughout for consistency

 - Consider rephrasing ” One strategy reported was to use lay advisors from the community to help with recruitment, wh *ich Douglas ( Douglas et al., 2011) found that community engagement* and lay advisors were the best method of recruitment of the South Asian community in their trial.” as current wording is unclear (typo?)

 - consider re-phrasing “We are involving PPI” to people with lived experience? aside from different terminology as noted above, the 'I' in PPI already covers 'involving'

Are the rationale for, and objectives of, the Systematic Review clearly stated?

Yes

Is the statistical analysis and its interpretation appropriate?

Not applicable

Are sufficient details of the methods and analysis provided to allow replication by others?

Yes

Are the conclusions drawn adequately supported by the results presented in the review?

Yes

Reviewer Expertise:

Clinical trials, operations, trial conduct

I confirm that I have read this submission and believe that I have an appropriate level of expertise to confirm that it is of an acceptable scientific standard.

# Reviewer report: Reviewer response for version 1

**DOI:** 10.3310/nihropenres.14679.r31813

**Published:** 2024-09-17

**Authors:** Vicki S Barber

**Affiliations:** 1 University of Oxford, Oxford, England, UK

Thank you for the opportunity to review this article. 

 This is an important topic for trialists across the UK and Ireland and worldwide.  Robust evidence and reporting of interventions to increase representation of underserved groups should be more prominent from grant development.  Some UK/Irish funders are now explicitly asking about how trial teams proposed to ensure better representation within trials and hopefully the indexing that result from those trials will add to the evidence base.  It would be useful for this important area to become a routine heading in protocols to ensure underrepresented populations are considered and how they could be better represented in trials, and then also subsequently reported.  

 There are a couple of typological issues that I would like to highlight to the authors that they might wish to consider - however the paper is scientifically sound and these changes will not affect materially the paper.

 Page 5 - Barriers section - should the cited paper not read as Corbie-Smith et al 2022 rather than Corbie-smith et al 2022 - both in this section and the references section. 

 Page 5 - Trial design section - it would perhaps be useful to the reader to have the Trial Forge website link not just a citation. 

 Page 5 - Strategies for improving inclusion in trials - first paragraph - consider if add in the paper to the others listed from the literature - Waheed et al., Recruitment and methodological issues in conducting dementia research in British ethics minorities: A qualitative systematic review.  Int J Methods Psychiatr Res. 2002:29:e1806

 Page 6 - Information sources section - In your ACCESS protocol you state your searches would be conducted using PubMED, ORRCA and the NIHR Journals Library.  I can see reference in the article to PubMED and ORRCA but no mention of the NIHR Journals Library - did you deviate from your protocol?

 Page 16 - Evaluation of methodological interventions to improve recruitment of underserved groups section - bottom of first paragraph - I think this is the first and only time you have used the abbreviation SE.

 This paper will be an important prompt to the trials community in their addressing of inequalities and ensuring the ability for far better and wider inclusion in the trials that are undertaken.

Are the rationale for, and objectives of, the Systematic Review clearly stated?

Yes

Is the statistical analysis and its interpretation appropriate?

Not applicable

Are sufficient details of the methods and analysis provided to allow replication by others?

Yes

Are the conclusions drawn adequately supported by the results presented in the review?

Yes

Reviewer Expertise:

Trial operations

I confirm that I have read this submission and believe that I have an appropriate level of expertise to confirm that it is of an acceptable scientific standard.

## References

[1] : Recruitment and methodological issues in conducting dementia research in British ethnic minorities: A qualitative systematic review. *Int J Methods Psychiatr Res* .2020;29(1) : 10.1002/mpr.1806 e1806 10.1002/mpr.1806 31808215 PMC7051842

# Reviewer report: Reviewer response for version 1

**DOI:** 10.3310/nihropenres.14679.r31658

**Published:** 2024-09-15

**Authors:** Eleanor Mitchell

**Affiliations:** 1 University of Nottingham, Nottingham, England, UK

Thank you for asking me to review this paper which reports a scoping review of interventions to increase representation of under-served groups in RCTs. The topic is of high relevance to anyone with an interest in clinical trials and the authors should be commended for their work. The review was conducted to a high standard in line with guidelines for scoping studies, prospectively registered and the protocol made publicly available.  The paper was very well written and a pleasure to read.  The Plain English Summary was excellent. The comments I make below are all minor but hopefully useful to further enhance the paper.

**Introduction**

- “There are several negative consequences to participant populations that do not look like the patients that could ultimately receive the trial’s intervention.” I understand what you’re saying here but think it would be better to state “….populations that are not the same as the patients that could….”
- Trial design: Give reference or weblink to Trial Forge website.

**Methods**

- Quality of included studies. Authors state they did not perform a formal quality assessment of papers. Could they briefly comment why?

**Results**

- Table 3: Forster et al, 2010. In the “ intervention to improve recruitment” column, the sentence appears incomplete.

- Table 3: Recruitment approach. Agnew et al 2013. In Author’s findings, its stated that authors reported their strategy was successful. Can you briefly add a statement to suggest why or was this data unavailable?
- Evaluation of methodological interventions to improve recruitment of under-served groups, 2 ^nd^ paragraph. “…this did not affect the age of those responding”. I think the authors mean that the £100 incentive did not increase participation of older people, but this could be made clearer.

**Discussion**

- Strengths and limitation, 2 ^nd^ paragraph: “as there is a work relating to….”. The ‘a’ should be removed. 4 ^th^ paragraph, give acronym for PPI in the first sentence.
- The first sentence in the ‘recommendations/future research’ could be made clearer.

Are the rationale for, and objectives of, the Systematic Review clearly stated?

Yes

Is the statistical analysis and its interpretation appropriate?

Not applicable

Are sufficient details of the methods and analysis provided to allow replication by others?

Yes

Are the conclusions drawn adequately supported by the results presented in the review?

Yes

Reviewer Expertise:

Clinical trial design and conduct

I confirm that I have read this submission and believe that I have an appropriate level of expertise to confirm that it is of an acceptable scientific standard.
